# *Tbx1* Heterozygosity in the Oligodendrocyte Lineage Shifts Myelinated Axon Composition in the Mouse Fimbria Without Behavioral Impairments

**DOI:** 10.21203/rs.3.rs-9327970/v1

**Published:** 2026-04-19

**Authors:** Anne Marie Wells, Takaki Tanifuji, Takeshi Takano, Arumu Endo, Gina Kang, Marisa Esparza, Qian Shi, Manzoor A. Bhat, Noboru Hiroi

**Affiliations:** University of Texas Health Science Center; University of Texas Health Science Center; University of Texas Health Science Center; University of Texas Health Science Center; University of Texas Health Science Center; University of Texas Health Science Center; University of Texas Health Science Center; University of Texas Health Science Center; University of Texas Health Science Center

**Keywords:** Tbx1, Pdgfrα, 22q11.2 CNV, social behavior, cognition, fimbria, axon, myelination

## Abstract

Constitutive heterozygosity of *Tbx1*, a T-box transcription factor gene in the 22q11.2 deleted region, produces behavioral deficits and alters myelinated axon composition in the mouse fimbria. However, the cellular origins of these effects—and whether axon changes causally drive behavioral impairments—remain unclear. Prior data link *Tbx1* heterozygosity to reduced oligodendrocyte precursor cell (OPC) markers in the fimbria in mice, raising the hypothesis that *Tbx1* deficiency specifically in the oligodendrocyte lineage contributes to myelin and behavioral phenotypes. To test this hypothesis, we first showed via *in vitro* siRNA knockdown that *Tbx1* regulates both OPCs and mature oligodendrocytes. We then generated conditional *Pdgfrα*Cre;*Tbx1*+/flox mice to initiate *Tbx1* heterozygosity in OPCs. These mice exhibited Cre-mediated recombination in *Pdgfrα*-expressing brain regions and OPC progeny in the fimbria. At 1 month of age, male mutants displayed enhanced spontaneous alternation in the T-maze relative to wild-type littermates—an effect absent at 2 months. No differences appeared in neonatal ultrasonic vocalizations, social interaction, novel object approach, anxiety-like behavior (elevated plus maze), or open-field locomotion and thigmotaxis. Electron microscopic analysis demonstrated a compositional shift in myelinated axons within the fimbria of adult male mutants: increased numbers in the 300–800 nm diameter range and decreased numbers at ~ 1,200 nm and ~ 1,400 nm, with unchanged myelin thickness across diameters. These results demonstrate that *Tbx1* heterozygosity in the oligodendrocyte lineage drives a selective shift toward smaller myelinated axons in the fimbria and a transient cognitive enhancement but does not recapitulate the full myelination abnormalities or the broader cognitive/social deficits observed in constitutive *Tbx1* heterozygotes. Thus, *Tbx1* function in non-oligodendrocyte lineage cells likely exerts non-cell-autonomous effects on myelination that contribute to neurodevelopmental behavioral impairments.

## Introduction

Cognitive and social impairments serve as predictors of mental illness(1, 2). Infants later diagnosed with autism spectrum disorder (ASD) exhibit delays and deviations in various aspects of motor, social, and language development(3–11). Similarly, children who later develop full-scale symptoms of schizophrenia show delays in the development of social cognition, working memory, attention, and processing speed(12–16), and these deficits remain integral to the disorder following its onset(17, 18).

These dimensional deficits in mental illness have genetic underpinnings. Copy number variations (CNVs)--which are deletions or duplications of up to several million base pairs at specific chromosomal loci encompassing numerous protein-coding genes–present high odds ratios and penetrance rates for cognitive and social deficits(19, 20) as well as mental illnesses(21–23).

Individuals carrying 1.5Mb to 3.0Mb hemizygous deletions of human chromosome 22q11.2 exhibit cognitive, social, and emotional impairments from childhood (24–26) and are diagnosed at elevated rates with anxiety disorders, attention-deficit hyperactivity disorder, ASD, intellectual disability, and schizophrenia(27, 28). Duplication of 22q11.2 is associated with epilepsy, ID, ADHD, and ASD at rates higher than those of non-carriers(29–45). However, the mechanisms by which how more than 40 protein-coding genes in 22q11.2 contribute to dimensional deficits and clinical diagnoses remain poorly understood in humans.

Several rare inherited cases of variants in the human *TBX1* gene, a T-box transcription factor gene located within the 22q11.2 CNV, are associated with ASD, schizophrenia, and intellectual disability in the absence of 22q11.2 CNVs(46–51). However, due to their rarity, the statistical reliability of the association between *TBX1* variants and mental illness is limited. Moreover, these patients often carry variants in other single genes throughout the genome(49), complicating the establishment of causality from these associations.

The deletion or overexpression of small murine chromosomal regions or single genes orthologous to human chromosome 22q11.2 offers a complementary approach (1, 52–56). Constitutive overexpression of several hundred kilobase pair segments of the mouse ortholog of 22q11.2 recapitulates distinct social and cognitive deficits linked to 22q11.2 duplication(52, 57), suggesting the presence of driver genes within this segment responsible for distinct behavioral phenotypes. *Tbx1* is one of the genes within a 200 kb segment of 22q11.2, overexpression and hemizygous deletion of which result in deficits in social behavior, repetitive behavior, and prepulse inhibition (50, 52).

The heterozygous deletion of *Tbx1* alone results in impairments in neonatal social communication(58–60), as well as post-pubertal and adult deficits in social interaction(58), social incentive learning(61), acoustic, but not non-acoustic, prepulse inhibition(50, 61), spatial memory acquisition in the Morris water maze and the speed to complete simple discrimination and reversal phases of attentional set-shifting, in the absence of non-specific deficits of motor speed (62). The fimbria of post-pubertal *Tbx1* heterozygous mice displays a lack of large myelinated axons, enhanced myelination of medium-sized axons, a compositional shift of myelinated axons to smaller sizes, and a gene expression profile suggestive of defective oligodendrocyte precursor cells(62). Furthermore, the volume of the secondary motor cortex and amygdala, regions involved in the vocal network(63), is reduced in post-pubertal *Tbx1* heterozygous mice (61). Additionally, virally induced overexpression of *Tbx1* in the hippocampus negatively impacts the developmental maturation of working memory capacity(64).

The cellular and developmental origins of the effects of *Tbx1* deficiency on oligodendrocyte precursor cells remain unknown. During the embryonic period, oligodendrocyte precursor cells emerge from embryonic neural progenitor cells in the medial ganglionic eminence of the ventricular zones around E12.5, followed by a second wave from the lateral ganglionic eminence at E15.5. Oligodendrocyte precursor cells continue to be generated from neural progenitor cells in the subventricular zone during the neonatal period (65–69).

Single-cell transcriptomic analyses have revealed that *Tbx1* is expressed in a more diverse array of cell types than previously indicated by *in situ* hybridization and immunocytochemical studies. In one analysis, involving 1.13 million cells from whole mouse embryos, *Tbx1* was detectable in mesodermal and ectodermal cells from E6.5 to E8.5, in ectodermal cells including those of the neural crest and neural tube at E8.25, in radial glial cells and oligodendrocytes at E10.5, and in neurons and oligodendrocytes at E10.5 and E12.5(70). In the mouse brain, *Tbx1* is detectable in cell clusters with signatures indicative of endothelial cells and Neurog2-positive radial glia at E18 and radial glial cells in the neonatal mouse brain(70). Similarly, another single-cell combinatorial indexing (sci)-RNA-seq analysis of approximately 2 million cells from mouse embryos at E9.5 to E13.3 identified *Tbx1* in endothelial cells, various types of neurons and their progenitors, neural progenitor cells, neural tube, and oligodendrocyte progenitor cells(71). Given that single-cell transcriptomic analyses are capable of detecting rare or transitional cell types with large numbers of cells, these studies suggest that *Tbx1* is present in radial glial cells and neural progenitor cells, as well as their progeny

Our previous work demonstrated that *Tbx1* heterozygosity initiated in neural progenitor cells during the neonatal period recapitulates some behavioral deficits associated with constitutive *Tbx1* heterozygosity(72). As neural progenitor cells during the neonatal period give rise to oligodendrocyte precursor cells, oligodendrocytes, neural progenitor cells, and neurons(65–69), we sought to determine whether the effects of *Tbx1* heterozygosity within the oligodendrocyte lineage induces behavioral and anatomical phenotypes. Our findings indicate that *Tbx1* heterozygosity in the oligodendrocyte lineage induces a compositional shift in myelinated axons in the fimbria, while no affecting myelin integrity, large myelinated axons, or behavior. These results suggest a more critical role for *Tbx1* in neurogenesis lineage.

## Methods

### Mice

All protocols for animal handling and use were approved by the Institutional Animal Care and Use Committee (IACUC) of the University of Texas Health Science Center at San Antonio (UTHSCSA) in accordance with National Institutes of Health (NIH) guidelines. Mice were housed in a vivarium with a standard light phase from 7 am to 9 pm).

C57BL/6J mice: We used 1- and 2-month-old male C57BL6/J mice (Jax #000664, Jackson Laboratory, Bar Harbor, ME) as stimulus mice for social interaction.

Pdgfrα Cre;Tbx1^+/flox^ mice: To conditionally induce *Tbx1* heterozygosity in oligodendrocyte precursor cells, we chose a *Pdgfrα*Cre line that initiates Cre synthesis earlier than a *Cspg4*Cre line to better induce recombination in the oligodendrocyte lineage(73). The *Pdgfrα-* promoter drives Cre-based recombination most robustly in oligodendrocyte precursor cells (74), but also in astrocytes, neurons, fibroblast-like cells, endothelial cells, vascular and leptomeningeal cells, and pericytes in the mouse brain(74–77).

We crossed hemizygous *Pdgfrα*Cre mice with *Tbx1*^+/flox^ mice to generate *PdgfrαCre;Tbx1*^+/flox^ mice. *PdgfrαCre* breeder mice (C57BL/6-Tg(*Pdgfrα*-Cre)1Clc/J; Jax #013148, Jackson Laboratory, Bar Harbor, ME) have a mixed C57BL/6J;C57BL/6N background. We backcrossed non-congenic *Tbx1*^+/flox^ mice(78) to C57BL/6J for more than 10 generations and confirmed that there was Cre-dependent recombination in a congenic *Tbx1*^+/flox^ mice(72). The genetic background of *Pdgfrα*Cre and *Tbx1*^+/flox^ lines were mixed C57BL/6J;C57BL/6N and C57BL/6J, respectively. These two C57BL/6 substrains have many behavioral differences(79). To avoid the confounding effects of their unequal genetic backgrounds(80), we used only an F1 generation derived from crosses of the *Pdgfrα*Cre and *Tbx1*^+/flox^ lines.

Pdgfrα Cre;ROSA-tdTomato mice: We crossed hemizygous *Pdgfrα*Cre mice (C57BL/6-Tg(*Pdgfrα*Cre)1Clc/J; Jax #013148, Jackson Laboratory, Bar Harbor, ME) with homozygous congenic *ROSA-CAG-tdTomato* mice (B6.Cg-*Gt(ROSA)26Sortm14(CAG−tdTomato)Hze*/J, Jax # 007914, Jackson Lab, Bar Harbor, ME) to generate *Pdgfrα*Cre;*ROSA-CAG-tdTomato* mice. This ROSA line expresses tdTomato in the hippocampus without the action of Cre (http://connectivity.brain-map.org/transgenic/experiment/81560256)

We determined the genotypes of the mice using our previously published protocol with primers shown in Table S1. The sex of the mice was determined by inspection of their external genitalia.

### In vitro cell culture of oligodendrocytes

Lateral ventricle tissues, including the subventricular zone (SVZ), were obtained from postnatal day 1–2 (P1–2) C57BL/6J pups. Cells were isolated in culture, with each culture derived from a single mouse. The cells were cultured in a medium (DMEM/F12, HEPES [11320–032, Gibco, Grand Island, NY, USA]) supplemented with N2 (17502048, Gibco, Grand Island, NY, USA), B27 (17504044, Gibco, Grand Island, NY, USA), epidermal growth factor (EGF) at a concentration of 20 ng/ml (AF100–15, Peprotech, Cranbury, NJ, USA). After two to four passages, the cells were dissociated from the spheres using StemPro Accutase Cell Dissociation Reagent (A1110501, Gibco, Grand Island, NY, USA). The cells were then divided into equal portions and plated on each Poly-L-ornithine (P4957, Sigma, St. Louis, MO, USA) and Bovine Fibronectin (1030-FN, R&D Systems, Minneapolis, MN, USA)-coated 6well plate (Corning, 351146, Corning, NY, USA,). The cells were cultured for 144 hours in medium with 5% fetal bovine serum after withdrawing EGF from the medium to induce and maintain differentiation. At the time of EGF withdrawal, we applied *Tbx1* siRNA (10nM, cat#4390771, s74767, Invitrogen; Thermo Fisher Scientific, Waltham, MA, USA) and control siRNA-A (10nM, cat#4390843, Invitrogen; Thermo Fisher Scientific, Waltham, MA, USA) together with siLentFect^™^ Lipid Reagent for RNAi, 0.5 ml (BIO-RAD, cat# 1703360, Hercules, CA, USA).

### Quantitative reverse transcription polymerase chain reaction (qRT-PCR)

In accordance with our published procedure(62), total RNA was extracted using an RNeasy Plus Mini Kit (Cat#74134, Qiagen, Germantown, USA). Complementary DNA (cDNA) was synthesized from total RNA using SuperScript IV VILO Master Mix (Cat#11766050, Invitrogen, Carlsbad, USA). qRT-PCR reactions were performed in triplicate on a QuantStudio 6 Flex Real-Time PCR System (Cat#4485694, Applied Biosystems, Waltham, USA) using the TaqMan Fast Advanced Master Mix (Cat#4444963, Applied Biosystems, Waltham, USA). We used TaqMan^®^ Gene Expression Assays (cat# 4331182, Thermo Fisher, Waltham, MA, USA). The Taqman probes are listed in the Supplementary Material (Table S2). Data were analyzed using the ΔΔCt method and normalized to the reference gene *Pgk1*; we had an identical pattern of results with another reference gene *18S*.

### Whole brain tissue clearing and index matching

We used the SHIELD and SmartBatch+ (LifeCanvas Sciences, Cambridge, MA) standard pipelines for preserving and electrophoretic clearing(81) to visualize tdTomato in the brains of *Pdgfra*Cre;ROSA-CAG-tdTomato mice. We performed transcardial perfusion of 1-month old mice with ice-cold 0.9% NaCl in dH_2_O, followed by 4% paraformaldehyde (PFA) in phosphate-buffered saline (PBS). Brains were preserved using SHIELD as follows: perfused brains were 1) removed from the cranium and fixed in 4% PFA in PBS overnight at 4°C with gentle shaking; 2) incubated in SHIELD OFF solution (dH_2_O + SHIELD Buffer [cat#SH-Bf] + SHIELD Epoxy Buffer [cat#SH-Ex]) solution at 4°C with gentle shaking for 3 days; 3) incubated in pre-warmed SHIELD ON Buffer (cat#SH-ON) at 37°C for 1 day with gentle shaking; 4) incubated in the delipidation buffer (cat#DB) for 3 days at 45°C with gentle shaking. Final clearing by electrophoresis was performed with the SmartBatch+ device, with the delipidation buffer inside the clearing cup containing the samples and conduction buffer (cat#CB) for 30 h. Cleared brains were index-matched using a solution of 50% EasyIndex (RI = 1.52, cat#EI-500–1.52) + 50% dH_2_O with shaking at 37°C for 1 day, then 100% EasyIndex for one additional day until brains were transparent(81).

### Light sheet microscopy

We used a Zeiss Lightsheet 7 Microscope housed in the UTHSCSA Optical Imaging Core to perform volumetric light sheet imaging of cleared *Pdgfrα*Cre;ROSA-CAG-tdTomato brains using a pair of 5×/0.1 focusing illumination objectives and a Fluar 2.5×/0.12 detection objective at identical settings. Image tiles were stitched together using Zeiss Zen Blue software (ver 3.4). 3D volume analysis was performed with Imaris (Oxford Instruments, ver 10.02). Peak intensity of the tdTomato signal was segmented, and volumetric measurements (mm^3^) and peak intensities of the total volume (arbitrary units) were calculated for each volume in each mouse.

### Immunofluorescence

*Pdgfrα*Cre;ROSA-*tdTomato* mice were sacrificed at 1 month of age. Mice were deeply anesthetized using 4–5% isoflurane and perfused transcardially with 0.9% saline followed by 4% PFA. Brains were extracted and post-fixed in 4% PFA overnight at 4°C, followed by cryoprotection in glycerol overnight at 4°C. Coronal 60-μm thick sections were cut with a freezing microtome. For immunofluorescence labeling, sections were washed in 0.1 M PBS, blocked with 5% normal donkey serum in PBS, and incubated overnight at room temperature, as we described previously(62, 72). We used a well-validated primary rabbit antibody against myelin basic protein (1:200, MBP, ab40390, Abcam) and Donkey anti-rabbit IgG-Alexa Fluor 488 (1:200, A21206, Invitrogen), together with a nuclear marker (DAPI,1:25,000, D3571, Invitrogen). MBP is a marker of mature oligodendrocytes(82, 83). We captured images of sections that were mounted and cover-slipped with Vectashield^®^ anti-fade mounting medium (H-1000) using a Keyence microscope (BZ-X800) microscope with BZX Hardware Module and Zeiss LSM 710 confocal microscope at the UT Health Optical Imaging Core.

### Double staining of tdTomato with markers of oligodendrocytes

We captured a series of coronal brain sections from *Pdgfrα*Cre;*ROSA-tdTomato* mice using a Keyence BZ-X800 microscope with BZX Hardware Module and a Zeiss LSM 710 confocal microscope at the UT Health Optical Imaging Core. We used the DAPI signal to identify the anatomical boundaries of landmark structures in each image, cross-matched with equivalent levels in the Allen Brain Atlas (Allen Reference Atlas – Mouse Brain [brain atlas]. Available from atlas.brain-map.org). We then used a color scale to represent the level of tdTomato intensity within the delineated anatomical boundaries.

### Behavioral Analysis

Male neonatal mice were tested for vocalization resulting from maternal separation at P8 and P12, followed by additional behavioral tests at 1 and 2 months of age, corresponding to peripubertal and post-pubertal, adolescent periods(84), respectively; early signs of puberty begin around 1 month of age(85). Because body weight can alter behavior in mice(86) and *Pdgfrα* regulates progenitor differentiation into adipocytes(87, 88), we measured body weight at the beginning of behavioral tests. Our behavioral battery included reciprocal social interaction, novel object approach, spontaneous alternation in a T-maze, elevated plus maze, and locomotor activity and thigmotaxis in an inescapable open field(52, 57–59, 64, 89–91).

The order of the behavioral assays was based on the stress level; tasks that in a home cage-like setting were given first (i.e., social interaction and novel object approach). T-maze and elevated plus maze tests permit choices and are thus considered less stressful than an inescapable open field. At least a one-day interval was provided between behavioral tests to reduce the likelihood of carryover effects(61, 62, 92), except for the T-maze, in which three different delays were given on three consecutive days.

Mice were assigned randomly to experimental groups and tested during the light phase. Experimenters were blinded to genotypes. Social interaction, novel object approach, and behaviors in the elevated plus maze were recorded using a Basler GigE camera, with video images stored in Ethovision v18 software (Noldus Information Technology, Leesburg, VA). Video images of social interaction and novel object approach were manually scored according to our established criteria. Various parameters of the elevated plus maze were automatically analyzed by Ethovision. The time point of each arm entry was recorded for spontaneous alternation, and the data were subsequently tallied manually. Locomotor activity and thigmotaxis in an inescapable open field were analyzed using Med Associates Activity Monitor 7 Software (Fairfax, VT).

Ultrasonic vocalization Pups were tested for vocalization induced by maternal separation at postnatal days 8 and 12. A cage containing the mother and a litter was transferred to the test room 30 min before testing. Ultrasonic vocalization was recorded for 5 min by UltraSoundGate (Avisoft, Germany) connected to a computer equipped with Avisoft-RECORDER software (Avisoft, Germany) in a test chamber (18 cm long × 18 cm wide × 30 cm high). The sampling rate and lower cutoff frequency were set at 250 kHz (format, 16-bit) and 10 kHz, respectively. A frequency window from 15–150 kHz was used for analysis. Call detection was provided by an automatic threshold-based algorithm and a hold-time mechanism (hold time = 10 ms). Sonograms were inspected, and mechanical noises were eliminated from the analysis. We used the VocalMat software, which has the lowest false positive and false negative rates(93), to determine call types. This software typically detects only the most salient components of the harmonic call type and classifies this call type differently. As the harmonic call type is often affected in genetic mouse models of neuropsychiatric disorders(58, 59, 86, 94), we manually inspected all call types and re-classified such cases as harmonic calls.

Reciprocal Social Interaction Test A test mouse and an age-matched unfamiliar C57BL/6J mouse as a stimulus subject were simultaneously placed in a cage (28.5 cm long × 17.5 cm wide × 12.5 cm high). Mouse behavior was recorded during two 5-min sessions with a 30-min interval between sessions. Video images were scored manually for affiliative and aggressive social interaction by a rater blinded to genotype. The following reciprocal social behaviors were scored by the duration of interaction (minimum of 1 s): aggressive (tail rattle, bite/kicks, sideway offense, boxing/wrestling), affiliative, non-aggressive (mount, pursuit, olfactory investigation, allogrooming, escape/leap), or passive (side-by-side, submissive). We analyzed the sum of time engaged in affiliative, active, and passive social interactions, but passive behavior was rarely seen in our experimental set-up (72, 86, 91, 94).

Novel Object Approach A test mouse was placed in a cage (28.5 cm long × 17.5 cm wide × 12.5 cm high) with an empty, non-secured 50-mL Falcon tube (3 cm diameter × 8.5 cm long) lying on its side. Video images of mouse interactions with the tube were used to manually score approach behavior by two independent analysts blinded to genotype with an inter-rater reliability of > 99%. Approach behavior was scored for olfactory or non-olfactory investigations with a minimum duration of 1 s. Time spent near the tube was also analyzed.

Spontaneous Alternation in a T-Maze A test mouse was placed in the start site of the long arm of a black plexiglass T-maze (21.5 cm long × 10.5 cm wide × 20.5 cm high) and allowed to enter the maze and explore either the left or right short arms (31 cm long × 10.25 cm wide × 20.5 cm high) of the cage. We imposed 0 s, 15 s, or 30 s interval delays to maze reentry for ten trials, and the three interval trials were given on three consecutive days. We manually scored the percentages of alternation and latency to reach the alternated and non-alternated arms.

Elevated Plus Maze Test Mice were placed in the center stage (5 × 5 cm) of the maze apparatus with four arms (30 × 5 cm) and permitted to explore two closed arms and two open arms extending from the center platform of the maze for 5 min. The maze was positioned 53 cm above the floor. The time spent in visits and the frequency of visits to the two open and two closed arms were analyzed.

Open Field Mice were placed in an open-field cage (27.3 cm × 27.3 cm × 20.2 cm; Med Associates, Fairfax, VT) and permitted to explore the cage for 30 min. We measured the distance and velocity of motor activity and duration of time spent in the center (19.05 cm × 19.05 cm central square) versus the margin of an open field, using the activity monitor software (Med Associates, Fairfax, VT).

### Electron Microscopy (EM)

We prepared tissue for EM to characterize myelination in the fimbria of *Pdgfrα*Cre;*Tbx1*^+/flox^ mice as we described previously(62). After behavioral assays were completed at 1 and 2 months of age, 4–5-month-old male *Pdgfrα*Cre;*Tbx1*^+/flox^ (N = 3) and five control mice, including *Pdgfrα*Cre;*Tbx1*^+/+^ (N = 2), wild-type (WT);*Tbx1*^+/flox^ (N = 2), and WT;*Tbx1*^+/+^ (N = 1), were anesthetized with 4.5% isoflurane in a chamber, and anesthesia was maintained by a nose cone with 2.0% isoflurane. The animals were transcardially perfused with 120 mL of 0.9% saline followed by fixation with 120 mL of 0.1 M buffer (pH 7.4; cat#11653 Electron Microscopy Science, Hatfield, PA) with 2.5% sodium glutaraldehyde (cat#16310, Electron Microscopy Sciences, Hatfield, PA) and 2.5% paraformaldehyde (cat#19202, Electron Microscopy Sciences, Hatfield, PA). Brains were extracted and post-fixed in the same solution at 4°C for 7 weeks. The fimbria from the two hemispheres were obtained separately using a vibratome and placed in 0.1 M sodium cacodylate buffer overnight. Tissues were rinsed with 0.1 M sodium cacodylate buffer to remove aldehydes and placed in 2% osmium tetroxide solution (OsO_4_; cat#19150, Electron Microscopy Sciences, Hatfield, PA) in 0.1 M sodium cacodylate buffer for 1 h. Tissues were dehydrated in a series of ethanol solutions and embedded in molds containing Polybed resin (Poly/Red^®^ 812 Embedding media, cat#08791 − 500 Polysciences, Inc., Warrington, PA). We cut 100-nm sections and collected them on square 150-mesh copper grids (cat#7551C, Polysciences, Inc., Warrington, PA) and stained them with uranyl acetate (7 g in 100 mL deionized water)/Reynold’s lead citrate (1.33 g lead nitrate,1.76 g sodium citrate in 30 mL triple-distilled water, 8 mL 1 N NaOH). Uranyl acetate stains membranous structures and structures containing nucleic acid; lead citrate binds to RNA-containing structures and the hydroxyl groups of carbohydrates.

Each fimbria section was viewed in the EM grid squares. Images were screened at 1,000× magnification. For each section, we used all grid images that contained fimbria tissue with round axons without wrinkles, folds, or tears in the sections. One image was captured at the center of each grid field at 20,000× magnification. Images were analyzed by MyelTracer(95). Only axons whose myelin was fully within the image were analyzed quantitatively.

We obtained multiple images for each animal from a section of the right and left hemispheres. The positions of sections sliced from the fimbria varied between the two hemispheres and did not necessarily match the two sides; in one case, only one hemisphere was available because of the accidental loss of a tissue slice. The number of images varied across tissue slices, resulting in unequal image numbers. Many axons from each grid square and many grid squares from each hemisphere were analyzed for each animal. As the number of images and positions of each image varied between the two hemispheres and did not match between the two sides, the hemisphere could not be used as a replicate. Thus, we pooled data for each animal as a technical replicate; data points within and across images were technical replicates. As there was considerable variability in the data, we could not average the values for each animal. The averages of the total axon diameter per genotype were also not appropriate for this data set, as axon numbers differed between genotypes at specific axon diameters (≥ 300 nm to < 400 nm; ≥1,200 nm to < 1300 nm) (genotype × axon diameter range, F(27,5880) = 6.336, *P* < 1.0 × 10^− 4^). Thus, linear mixed models were used, as reported previously(62). Data were assessed for the number of myelinated axons, the thickness of myelination, and the g-ratio along a 100 nm unit axon diameter. Data from different images and hemispheres were embedded in random models.

### Statistical Analysis

We used SPSS (v29.0.2.0 (20), IBM Corporation) to perform all statistical analyses. Among-group and between-group comparisons of the data were performed using analysis of variance (ANOVA) and Student’s two-tailed t-test (α = 0.05). We determined normality and homogeneity of variance using the Shapiro-Wilk test and Levene’s homogeneity of variance test, respectively If either assumption was violated, we analyzed the data using a linear mixed model, Kruskal Wallis tests, or Mann–Whitney U tests. The Greenhouse–Geisser correction was applied if sphericity was violated and the estimated epsilon was less than 0.75. If multiple tests were applied to a data set, the significance level was adjusted using the Benjamini–Hochberg correction, with a false discovery rate of 5%. We used GraphPad Prism software (v9; GraphPad Software, San Diego, CA) to generate all graphs. All statistical analyses are presented in Table S3.

### Data Availability

All data that support the findings and conclusions are provided within the article. All raw data and additional information are available upon request.

## Results

### Effects of Tbx1 Knockdown on Markers of Oligodendrocyte Lineage In Vitro

We first determined the earliest step along the oligodendrocyte lineage at which *Tbx1* deficiency has an effect. To this end, we developed an *in vitro* screening assay that capitalizes on the capacity of neonatal neural progenitor cells, derived from the subventricular zone of C57BL/6J pups sacrificed on postnatal day 2, to generate neuronal precursor cells and oligodendrocyte precursor cells(69). We evaluated the expression of *Cspg4*, a marker of oligodendrocyte precursor cells, as well as *Mag, Mbp, Mog*, and *Plp1*, which are markers indicative of maturing and mature myelinating oligodendrocytes (65), at 144 hours after EGF withdrawal (i.e., differentiation) and *Tbx1* siRNA application.

**Figure 1:**
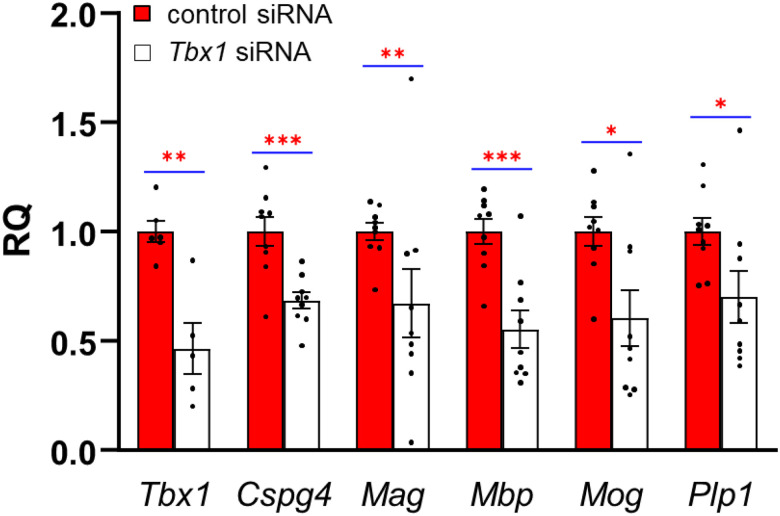
The relative quantification (RQ) (mean ± SEM) of expression levels at each stage of oligodendrogenesis, as determined by quantitative reverse transcription polymerase chain reaction (qRT-PCR) using *Pgk1* as a reference gene. Three cell clones were derived from three different C57BL/6J pups at postnatal days 2 or 3, with data obtained from three culture wells for each clone, except for one *Tbx1*siRNA-treated clone where *Tbx1* mRNA was undetectable and thus excluded from the analysis. A linear mixed model showed that Tbx1 siRNA reduced expression of all genes tested equally (Treatment, F(1,89)=14.665, p=0.0002386; Gene, F(5,89)=0.8440, p=0.5222; Treatment x Gene, F(5,89)=0.8440, p=0.5222). Mann-Whitney tests confirmed that *Tbx1* siRNA reduced expression of each gene. Statistically significant differences between control siRNA and *Tbx1* siRNA groups are indicated by *, **, and *** for p <0.05, p<0.01, and p<0.005, respectively; they remained significant following Benjamini-Hochberg correction at a 5% false discovery rate (FDR).

*Tbx1* siRNA significantly reduced mRNA levels of *Tbx1, Cspg4*, and all markers of mature oligodendrocytes, with *Pgk1* as (Fig. 1) and *18S* (**Table S3**-Figure 1) as reference genes. These *in vitro* findings are consistent with our *in vivo* data indicating that *Cspg4* (also known as *Ng2*) was reduced in the fimbria of *Tbx1* heterozygous mice(62).

As our culture includes both neuronal (58, 72) and oligodendrocyte (see Fig. 1) lineages, as well as remaining neural progenitor cells, how *Tbx1* knockdown induces changes in the expression of myelin marker genes remains unclear. *Tbx1* knockdown in oligodendrocyte precursor cells may reduce myelin markers in a cell-autonomous manner; alternatively, reductions in *Tbx1* in neonatal neural progenitor cells or their neuronal progeny may indirectly regulate these markers of oligodendrocytes.

### Localization of Recombination in Pdgfrα-Cre;ROSA-tdTomato Mice

**Figure 2:**
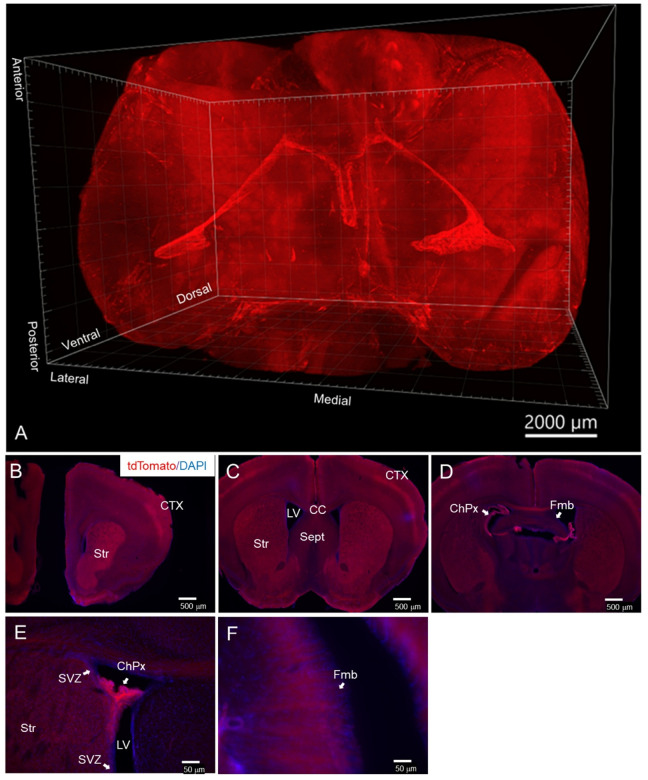
**A**. A representative lightsheet image of the brain from a 1-month-old male PdgfrαCre;ROSA-tdTomato mouse. Following SmartBatch+ tissue delipification, tdTomato signals were visualized using a Zeiss LSM 7 Lightsheet microscope with a 2.5x objective (N = 3).**B-D**. tdTomato expression in 1-month-old PdgfrαCre;ROSA-tdTomato mice is depicted together with blue DAPI signals at the levels of the anterior striatum (**B**), middle striatum (**C**), posterior striatum/fimbria (**D**), anterior lateral ventricle (**E**), middle lateral ventricle (**F**), and fimbria (**H**). Abbreviations: ChPx, choroid plexus; LV, lateral ventricle; Str, striatum; Sept, septum; CC, corpus callosum; CTX, cortex; HGCL, hippocampal granule cell layer; fmb, fimbria; SVZ, subventricular zone. N = 3.

Given our data suggesting that *Tbx1* deficiency begins to affect the stage of oligodendrocyte precursor cells (see Fig. 1), we initiated *Tbx1* heterozygosity in oligodendrocyte precursor cells using a conditional heterozygous mouse model, where Cre-based initiation of *Tbx1* heterozygosity is guided by the promoter of *Pdgfrα*, a gene expressed in oligodendrocyte precursor cells of both embryonic and post-embryonic origins. We first assessed the extent of *PdgfrαCre*-mediated recombination throughout the brain, employing a tissue clearing technique (SmartBatch+) and conducting lightsheet microscopy to visualize the 3D volume of tdTomato signals in the brains of PdgfrαCre;ROSA-tdTomato mice (Fig. 2A). The highest levels of tdTomato expression were observed in a pair of arch-shaped structures located along the lateral and third ventricles. Additionally, clusters of tdTomato signals were detected on the ventral surface of the posterior brain, while lower levels of signals were seen throughout other regions.

To identify brain regions with tdTomato signals in PdgfrαCre;ROSA-tdTomato mice, we analyzed a series of coronal sections from 1-month-old PdgfrαCre;ROSA-tdTomato mice. High levels of tdTomato signals were present in the striatum, superficial layers of the neocortex (Fig. 2BC), choroid plexus within the lateral ventricle (Fig. 2ED) and the fimbria (Fig. 2F). This expression pattern, driven by the *Pdgfrα* promoter, is consistent with previously reported distributions of *Pdgfrα* mRNA and protein, as well as recombination activity associated with the *Pdgfrα* promoter in the mouse brain (96–98). Moreover, the most intense staining of the choroid plexus is consistent with the intensely tdTomato+ structures resembling the shapes of the lateral and third ventricles (see Fig. 2A).

**Figure 3:**
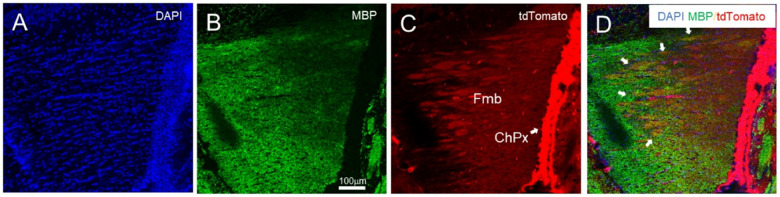
A Representative Case of Confocal Images of the Fimbria of a 1-Month-Old Male PdgfrαCre;ROSA-tdTomato Mouse. Sections were stained for DAPI (**A**, blue) and MBP (**B**, green).**C**. Red tdTomato signals originated from the PdgfrαCre;ROSA-tdTomato genotype.**D**. A composite image of the three colors is presented; white arrows indicate an area where fibers are positive for both MBP and tdTomato. N = 4. ChPx, choroid plexus; fmb, fimbria.

Embryonic Pdgfra-positive cells give rise to distinct pre-oligodendrocyte precursor cells by the perinatal period, but they exhibit similar profiles by postnatal and adult periods(65, 99). PdgfraCre is expected to induce recombination in oligodendrocyte precursor cells(74). Once tdTomato is expressed through recombination in the PdgfraCre;ROSA-tdTomato mouse line, it remains expressed in their progeny, such as mature oligodendrocytes and myelinated fibers. As myelination of the fimbria is selectively affected in constitutive *Tbx1* heterozygous mice(62), we examined whether tdTomato was colocalized with MBP, a marker of mature oligodendrocytes and myelinated fibers, in the fimbria(100). MBP-positive fibers were present throughout the fimbria (Fig. 3AB). TdTomato signals were more prominent laterally than medially (Fig. 3C) and was colocalized with MBP-positive fibers (Fig. 3D) in the fimbria. These data establish that tdTomato triggered by PdgfraCre in PdgfraCre;ROSA-tdTomato mice is present in myelinated fibers in the fimbria.

Intense tdTomato signals were present in the choroid plexus located laterally to the lateral surface of the fimbria (Fig. 2D). The cell types in the mouse choroid plexus that express *Pdgfra* include fibroblasts, mural cells, and endothelial cells in the embryo, and fibroblasts, macrophages from the post-embryonic period in the mouse brain; while gene profiles indicative of oligodendrocyte precursor-like cells are detectable during the embryonic period, their genuine location within the choroid plexus remains unclear (70, 101–103). The robust tdTomato signal observed in the choroid plexus at 1 month of age likely originates from some or all of these cell types.

### Effects of Tbx1 Heterozygosity in Oligodendrocyte Precursor Cells on Behaviors

The rate of growth could affect behavior; PdgfrαCre;*Tbx1*^+/flox^, termed o*Tbx1*^+/−^ mice, may exhibit developmental delays, and their phenotypes could reflect a delayed development compared to their wild-type littermates. However, neonatal o*Tbx1*^+/+^ and o*Tbx1*^+/−^ mice demonstrated indistinguishable increases in body weights (**Figure S1**).

To determine how *Tbx1* heterozygosity in oligodendrocyte precursor cells and their progeny affects behavior, mice were tested for social communication during the neonatal period and a battery of social, cognitive, anxiety-related, and motor behaviors at one and two months of age, as myelination peaks around the fourth to fifth postnatal week in rodents(104, 105) and mice reach adolescence around 2 months of age(84). Constitutive *Tbx1* heterozygous mice exhibit impairments in 1) neonatal social communication as early as P7, 2) social behaviors, 3) working memory/cognitive flexibility in a T-maze and attentional set shifting, and 4) affect-related behaviors in an inescapable open field, though not in an elevated plus maze(58–62) around adolescence.

We first measured differences among the three control mice: PdgfraCre;*Tbx1*^+/+,^ WT;*Tbx1*^+/flox^, and WT;*Tbx1*^+/+^. There was no significant difference among the controls for any behavioral measure, expect for three time points of total margin time in the open field (see **Table S3**-Figure 8B); therefore, we collapsed all the control genotypes into a single control group as *oTbx1*^+/+^ for comparison with *oTbx1*^+/−^ (70).

Constitutive *Tbx1*^+/−^ mice exhibit various neonatal, peri-adolescent, and postnatal behavioral phenotypes(58, 59). Therefore, we tested mice for neonatal ultrasonic vocalization on P8 and P12–P13 and performed additional tests with peripubertal (1 month) and adolescent (2 months) mice with 1–2 day intervals between tasks to mitigate potential carryover effects of prior testing(92).

**Figure 4:**
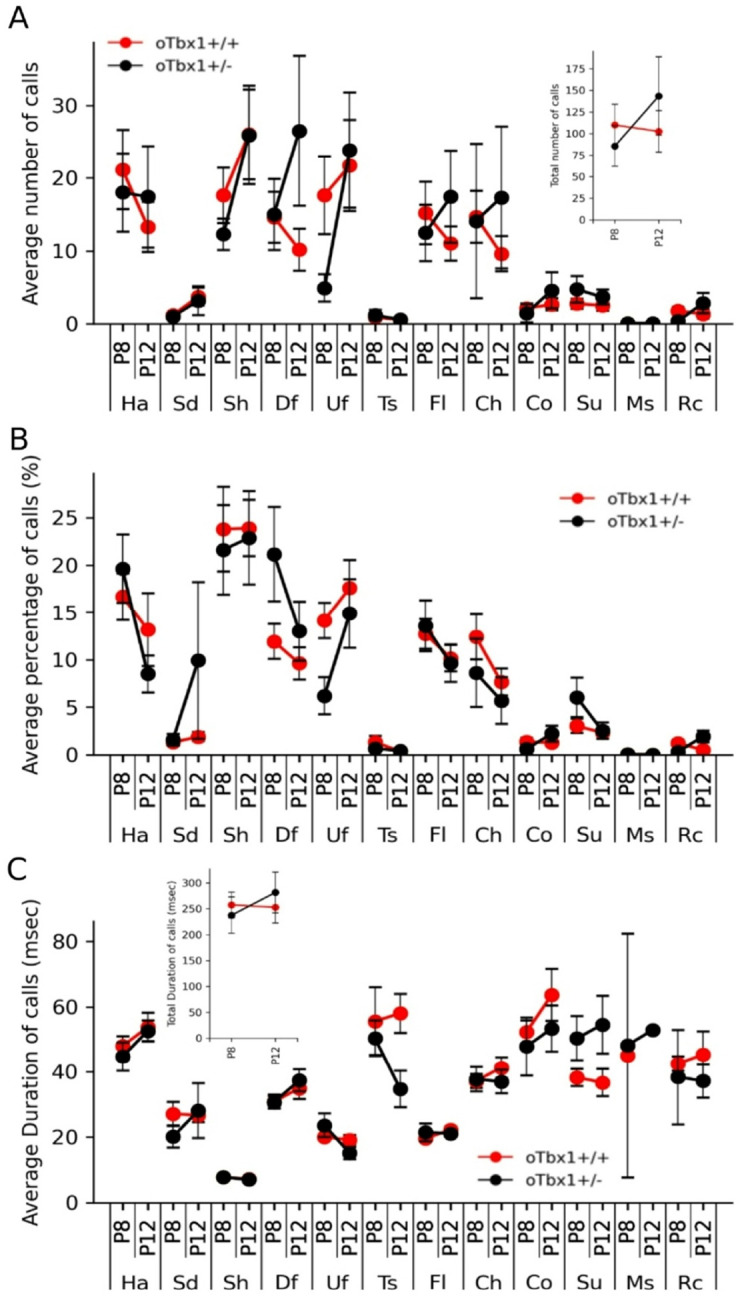
The number (**A**), proportion (**B**), and duration (**C**) (mean ± standard error of the mean [SEM]) of 12 call types emitted on postnatal days (P) P8 and P12. The two genotypes exhibited differences in certain interaction effects: **A.** Number: Genotype, F(1,912)=0.1966, p = 0.6576; Genotype x Postnatal day, F(1, 912)=4.835, p = 0.0281; Genotype x Call type, F(11, 9123)=0.7077, p = 0.7319; Genotype x Postnatal day x Call types, F(11, 912)=0.3686, p = 0.9679. **B**. Proportion: Genotype, F(1,912)=0.0107, p = 0.9176; Genotype x Postnatal day, F(1, 912)=0.0107, p = 0.9176; Genotype x Call type, F(11, 912)=1.3657, p = 0.1837; Genotype x Postnatal day x Call types, F(11, 912)=0.7966, p = 0.6437. **C**. Duration: Genotype, F(1,560)=0.0435, p = 0.8349; Genotype x Postnatal day, F(1, 560)=0.10519, p = 0.7458; Genotype x Call type, F(11, 560)=3.361, p = 0.00016; Genotype x Postnatal day x Call types, F(11, 560)=1.0389, p = 0.4101. However, Mann-Whitney U tests did not reveal any significant genotype effects at any postnatal day for any call type after applying Benjamini-Hochberg corrections at 5% false discovery rate (FDR). **Inset** in **A**: The total number of ultrasonic vocalizations (mean ± SEM) emitted at P8 and P12. The two genotypes did not differ on the two postnatal days (Genotype, F(1,76)=0.1559, p = 0.694; Genotype x Postnatal day, F(1, 76)=1.103, p = 0.2969). **Inset** in **C**: Duration: Genotype, F(1,32)=0.0002, p = 0.9878; Genotype x Postnatal day, F(1, 32)=0.3617, p = 0.5518). Ha, harmonic; Sd, step-down; Sh, short; Df, down frequency modulation; Uf, up frequency modulation; Ts, two steps; Fl, flat; Ch, chevron; Co, complex; Su, step-up; Ms, multiple steps; Rc, reverse chevron. *oTbx1*^+/+^ mice, N = 27; *oTbx1*^+/−^ mice, N = 11 at P8; *oTbx1+/+* mice, N = 30; *oTbx1*^+/−^ mice, N = 12 at P12.

**Figure 5:**
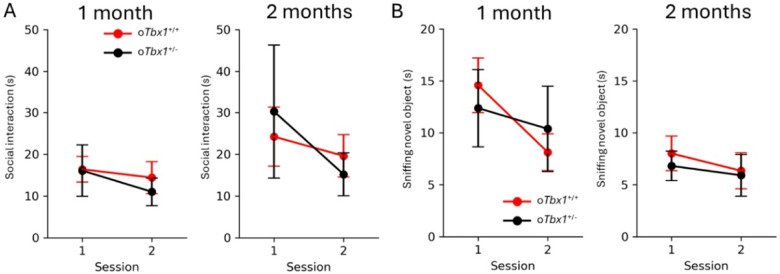
Interaction with Social and Non-Social Stimuli. A) Social Interaction. The *oTbx1*^+/−^ (PdgfrαCre;Tbx1^+/flox^) mice and *oTbx1*+/+ mice did not differ in the amount of time (mean ± SEM) spent in affiliative social interaction (1 month: genotype, F(1, 72) = 0.1118, p = 0.739; genotype × session, F(1, 72) = 0.262, p = 0.6102; 2 months: genotype, F(1, 58) = 0.00547, p = 0.9413; genotype x session, F(1, 58) = 1.2175, p = 0.2744). The three genotypes of *oTbx1*+/+ (1 month, N = 8, PdgfrαCre;*Tbx1*^+/+^, N = 10, Wild-type;*Tbx1*^+/+^, N = 9, Wild-type;*Tbx1*^+/flox^; 2 months, N = 8, PdgfrαCre;*Tbx1*^+/+^, N = 6, Wild-type;*Tbx1*^+/+^, N = 7, Wild-type;*Tbx1*^+/flox^) did not show significant differences (1 month: genotype, F(2, 48) = 1.686, p = 0.1960; genotype × session, F(2, 48) = 0.843, p = 0.4366; 2 months: genotype, F(2, 36) = 0.0392, p = 0.9616; genotype x session, F(2, 36) = 0.2382, p = 0.7893), and were thus combined as *oTbx1*+/+. *oTbx1*^+/+^ mice, N = 27; *oTbx1*^+/−^ mice, N = 11 at 1 month; *oTbx1*^+/+^ mice, N = 21; *oTbx1*^+/−^ mice, N = 10 at 2 months. **B**) Approach to a novel, non-social object. The two genotypes did not differ in the amount of time spent in affiliative social interaction (1 month: genotype, F(1, 70) = 2.2507E-05, p = 0.9962; genotype × session, F(1, 70) = 1.0305, p = 0.31354; 2 months: genotype, F(1, 58) = 0.11634, p = 0.7342; genotype × session, F(1, 58) = 0.0821, p = 0.77545). The three genotypes of *oTbx1*^+/+^ (1 month: N = 8, PdgfrαCre;*Tbx1*^+/+^, N = 9, Wild-type;*Tbx1*^+/+^, N = 9, Wild-type;*Tbx1*^+/flox^; 2 months: N = 8, PdgfrαCre;*Tbx1*^+/+^, N = 6, Wild-type;*Tbx1*^+/+^, N = 7, Wild-type;*Tbx1*^+/flox^) did not differ (1 month: genotype, F(2, 46) = 0.1772, p = 0.8382; genotype × session, F(2, 46) = 0.9318, p = 0.4012; 2 months: genotype, F(2, 36) = 0.0580, p = 0.9437; genotype × session, F(2, 36) = 0.6581, p = 0.5239) and were therefore combined as *oTbx1*^+/+^. *oTbx1*^+/+^ mice, N = 26; *oTbx1*^+/−^ mice, N = 11 at 1 month; *oTbx1*^+/+^ mice, N = 21; *oTbx1*^+/−^ mice, N = 10 at 2 months.

**Figure 6:**
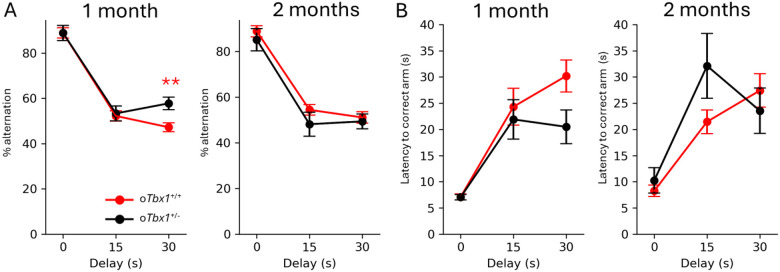
Spontaneous alternation in a T-maze. Compared to male *oTbx1*^+/+^ (N = 20) mice, male *oTbx1*^+/−^ (N = 10) mice performed better in the percentage of correct spontaneous alternations (mean ± SEM) at the longest delay (30 s) in a T-maze at 1 month of age, but not at 2 months. Specifically, at 1 month: % alternation, genotype, F(1, 96) = 4.66, p = 0.0333; genotype × delays, F(2,96) = 3.1509, p = 0.0473; at 2 months: genotype, F(1, 81) = 2.047, p = 0.1564; genotype × delays, F(2, 81) = 0.2439, p = 0.7841. The three genotypes of *oTbx1*^+/+^ (1 month: N = 8, PdgfrαCre;*Tbx1*^+/+^, N = 7, Wild-type;*Tbx1*^+/+^, N = 9, Wild-type;*Tbx1*^+/flox^; 2 months: N = 8, PdgfrαCre;*Tbx1*^+/+^, N = 6, Wild-type;*Tbx1*^+/+^, N = 6, Wild-type;*Tbx1*^+/flox^) did not differ (1 month: genotype, F(2, 63) = 0.1046, p = 0.9008; genotype × delays, F(4, 63) = 0.2648, p = 0.8995; 2 months: genotype, F(2, 51) = 0.1197, p = 0.8874; genotype × delays, F(4, 51) = 0.6401, p = 0.6363) and consequently were combined as *oTbx1*^+/+^. *oTbx1*^+/−^ mice exhibited higher levels of alternations at the 30-second delay compared to *oTbx1*+/+ mice, as determined by the Mann-Whitney test (p = 0.0081). **B**) The two groups did not exhibit significant differences in the latency to correct choices in the T-maze at 1 month (genotype, F(1, 96) = 2.181, p = 0.1430; genotype × delays, F(2, 96) = 2.058, p = 0.1332) or at 2 months (genotype, F(1, 81) = 1.1135, p = 0.2944; genotype × delays, F(2, 81) = 2.5825, p = 0.0817). The three genotypes of *oTbx1*^+/+^ (1 month, N=8), PdgfrαCre;*Tbx1*^+/+^ (N=7), Wild-type;*Tbx1*^+/+^ (N=9), and Wild-type;*Tbx1*^+/flox^ (2 months, N=8), PdgfrαCre;*Tbx1*^+/+^ (N=6), Wild-type;*Tbx1*^+/+^ (N=6), and Wild-type;*Tbx1*^+/flox^, demonstrated a significant interaction effect at 1 month of age (genotype, F(2, 63) = 1.5787, p = 0.2143; genotype × session, F(4, 63) = 4.5625, p = 0.00267), but no significant interaction at 2 months of age (genotype, F(2, 51) = 0.1631, p = 0.8500; genotype × session, F(4, 51) = 1.0611, p = 0.3853). However, Mann-Whitney U tests revealed that none of the three genotypes significantly differed from one another at 1 month of age after applying the Benjamini-Hochberg correction (p>0.05 for all pairs). The three genotypes were combined as o*Tbx1*^+/+^. The sample sizes were as follows: *oTbx1*^+/+^ mice, N = 24; *oTbx1*^+/−^ mice, N = 10 at 1 month; *oTbx1*^+/+^ mice, N = 20; *oTbx1*^+/−^ mice, N = 9 at 2 months.

We found no differences among o*Tbx1*^+/−^ pups in the number, percentage, and duration of various neonatal vocal call types on P8 and P12 (Fig. 4A–C). At 1 and 2 months of age, mice were sequentially tested for social interaction, novel object approach, spontaneous alternation in a T-maze, anxiety-related behavior in an elevated plus maze, and locomotor activity and thigmotaxis in an inescapable open field. *oTbx1*^+/−^ mice and o*Tbx1*^+/+^ mice were indistinguishable in social interaction (Fig. 5A) and novel object approach (Fig. 5B). In a T-maze, o*Tbx1*^+/−^ mice, at 1 month of age exhibited better spontaneous alternation rates at the longest delay (Fig. 6A); otherwise, o*Tbx1*^+/+^ and o*Tbx1*^+/−^ mice were indistinguishable in rates of spontaneous alternation at 2 months of age and in latencies to correct choices at 1 and 2 months of age (Fig. 6B–D).

**Figure 7:**
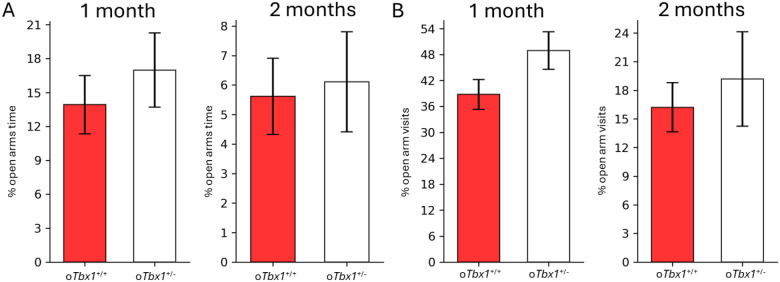
Anxiety-related behavior in an elevated T-maze. The *oTbx1*^+/−^ and *oTbx1*^+/+^ mice did not differ significantly in the relative amount of time spent (mean +SEM) in the open arms of an elevated plus maze (**A**,1 month,U = 100, p = 0.3440; 2 months, U = 98, p = 0.9483) or the frequency of visits to the open arms of the elevated plus maze (**B**, 1 month,t(32) = 1.7412, p = 0.0912; 2 months, t(28) = 0.5925, p = 0.5583). The three genotypes of *oTbx1*+/+ (1 month, N=7, PdgfrαCre;*Tbx1*^+/+^, N=7, Wild-type;*Tbx1*^+/+^, N=6, Wild-type;*Tbx1*+/flox; 2 months, N=8, PdgfrαCre;*Tbx1*^+/+^, N=6, Wild-type;*Tbx1*^+/+^, N=6, Wild-type;*Tbx1*^+/flox^) did not differ in the percentage of time spent in open arms (1 month,genotype, F(2, 20) = 0.0745, p = 0.9285; 2 months,genotype, F(2, 17) = 2.126, p = 0.1499) or the percentage of visits to open arms (1 month,genotype, F(2, 20) = 0.1837, p = 0.8336; 2 months,genotype, F(2, 17) = 1.4821, p = 0.2551), and thus were combined as *oTbx1*^+/+^. The sample sizes were: *oTbx1*^+/+^ mice, N = 23; *oTbx1*^+/−^ mice, N = 11 at 1 month; *oTbx1*^+/+^ mice, N = 20; *oTbx1*^+/−^ mice, N = 10 at 2 months.

The two genotypes were indistinguishable in the percentage of time spent in open arms (Fig. 7A) and visits to open arms (Fig. 7B) of the elevated plus maze.

**Figure 8:**
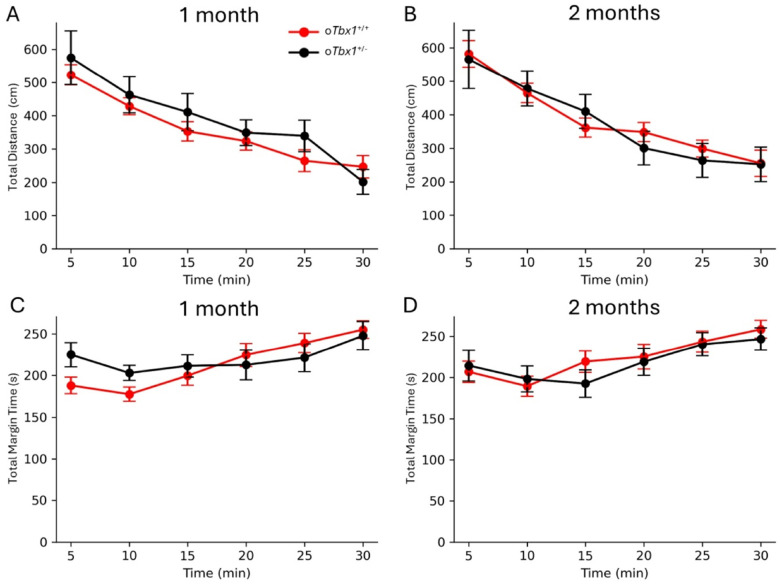
Motor Activity and Thigmotaxis. *oTbx1*^+/+^ and *oTbx1*^+/−^ mice exhibited indistinguishable distances traveled at both 1 month and 2 months of age (**A**, 1 month, genotype, F(1, 198) = 0.6314, p = 0.4278; genotype × time, F(5, 198) = 0.9551, p = 0.4466;**B**, 2 months, genotype, F(1, 28) = 0.0242, p = 0.87739; genotype × time, F(3.66, 102.485) = 0.5389, p = 0.69188). The Greenhouse–Geisser correction was applied due to a violation of sphericity, with the estimated epsilon being less than 0.75. The three genotypes of *oTbx1*^+/+^ (1 month, N=8, PdgfrαCre;*Tbx1*^+/+^, N=7, Wild-type;*Tbx1*^+/+^, N=9, Wild-type;*Tbx1*^+/flox^; 2 months, N=8, PdgfrαCre;*Tbx1*^+/+^, N=6, Wild-type;*Tbx1*^+/+^, N=6, Wild-type;*Tbx1*^+/flox^) demonstrated differences at both ages (genotype, F(2, 21) = 5.475, p = 0.01220; genotype × time, F(7.10, 74.58) = 1.118, p = 0.3611; at 2 months,genotype, F(2, 17) = 9.533, p = 0.00167; genotype × time, F(10, 85) = 0.3955, p = 0.9453). Comparisons of all pairs using Student’s t-tests indicated that no pair reached significance after the Benjamini-Hochberg correction (p>0.05 for all pairs), resulting in their combination as *oTbx1*^+/+^. *oTbx1*^+/+^ and *oTbx1*^+/−^ mice spent equal amounts of time in the margin zone (i.e., thigmotaxis) of the inescapable open field at both 1 and 2 months of age (**C,** 1 month, genotype, F(1, 198) = 0.2009, p = 0.6545; genotype × time, F(5,198) = 2.1516, p = 0.0609;**D**, 2 months, genotype, F(1, 168) = 0.08912, p = 0.7657; genotype × time, F(5, 168) = 0.9880, p = 0.4267). The three genotypes of *oTbx1*^+/+^ did not differ at 1 month of age (genotype, F(2, 21) = 0.3687, p = 0.6961; genotype × time, F(5.485, 57.58) = 0.9884, p = 0.4375), but differences were observed at 2 months of age (genotype, F(2, 17) = 4.880, p = 0.0211; genotype × time, F(5.049, 42.917) = 0.7179, p = 0.6147). Comparisons of all pairs using Student’s t-tests revealed that 2-month-old Wild-type;*Tbx1*^+/flox^ and Wild-type;*Tbx1*^+/+^ differed at the first four time points, even after the Benjamini-Hochberg correction (p<0.05). However, these groups had relatively small sample sizes (N=6 for both), and PdgfrαCre;*Tbx1*^+/+^and Wild-type;*Tbx1*^+/flox^ or PdgfrαCre;*Tbx1*^+/+^ and Wild-type;*Tbx1*^+/flox^ did not differ at any time point (p >0.05), thus they were combined as *oTbx1*^+/+^. Sample sizes included *oTbx1*^+/+^ mice, N = 24; *oTbx1*^+/−^ mice, N = 11 at 1 month; *oTbx1*^+/+^ mice, N = 20; *oTbx1*^+/−^ mice, N = 10 at 2 months.

We evaluated motor activity and anxiety-related behavior in the stressful open-field task(58, 62) where there is no opportunity to escape to closed space. In this task, o*Tbx1*^+/−^ and o*Tbx1*^+/+^ mice were indistinguishable in distance traveled at both 1 month and 2 months of age (Fig. 8A,B) and time spent in the margin zone (Fig. 8C,D).

While conditional *Tbx1* heterozygosity in the oligodendrocyte cell lineage resulted in improved spontaneous alternation with the longest inter-trial delay at one month of age, this phenotype contrasts with the impaired spontaneous alternation observed in constitutive *Tbx1* heterozygous mice (58, 62). Moreover, this conditional *Tbx1* heterozygosity did not replicate the altered neonatal vocalizations, peri-adolescent or postnatal social interaction deficits, or heightened responses to novel, non-social objects or thigmotaxis seen in constitutive *Tbx1* heterozygous mice(58).

### Effects of Tbx1 Heterozygosity in Oligodendrocyte Precursor Cells on Myelinated Axons in the Fimbria

After behavioral testing, we examined myelinated axons in the fimbria using electron microscopy. Constitutive *Tbx1* heterozygosity selectively alters the ultrastructure in the fimbria, resulting in a higher proportion of myelinated axons measuring 200–600 nm diameter and a lower proportion of axons ≥ 700 nm and < 1,200 nm diameters. Furthermore, the 700–1,500 nm axons exhibited a thicker myelin sheath, with no larger myelinated axons observed in constitutive *Tbx1* heterozygous mice (62). We evaluated whether the ultrastructural alterations of the fimbria of constitutive *Tbx1*^+/−^ mice were recapitulated in o*Tbx1*^+/−^ mice.

**Figure 9:**
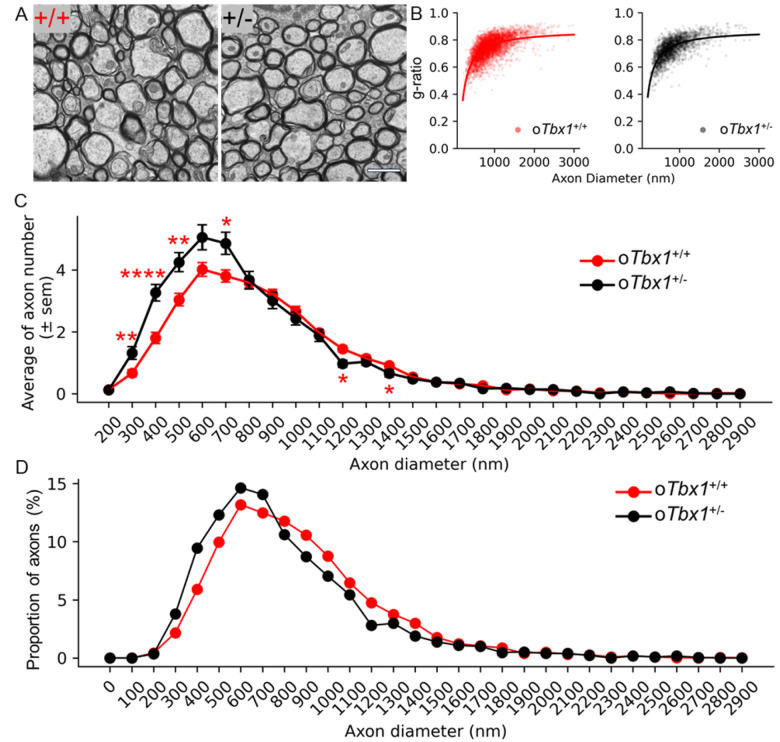
Electron Microscopy Analysis of Myelinated Axons.**A**. Representative electron microscopy images of axons in the fimbria at 20,000× magnification. Genotypes: +/+, *oTbx1*^+/+^; +/−, *oTbx1*^+/−^. Scale bar = 1,600 nm.**B**. G-ratios plotted against axon diameters for 4,423 and 2,317 axons from 5 *oTbx1*^+/+^ mice and 3 *oTbx1*^+/−^ mice, respectively. All axons with closed myelin sheaths were analyzed using MyelTracer. The reciprocal function best fits the data distributions. G-ratios increased as a function of axon diameter (*oTbx1*+/+, R = 0.6268, p < 0.001; *oTbx1*+/−, R = 0.6491, p < 0.001). There was no significant difference in the axon diameter range for G-ratios between oTbx1+/+ and oTbx1+/− mice (genotype, p = 0.8131; genotype × axon diameter, p = 0.221).**C**. Average number (± SEM) of axons per image field versus axon diameter. Significant differences were observed between *oTbx1*^+/+^ and *oTbx1*^+/−^ mice (c (27) = 109.7085, p = 6.22 × 10), primarily due to a higher number of axons with diameters ≥300 nm and <800 nm and a lower number of axons with diameters ≥1,200 nm and <1,500 nm in *oTbx1*^+/−^ compared to *oTbx1*^+/+^ mice, as determined by Mann-Whitney U tests applied to this diameter range (*, p < 0.05; **, p < 0.01; ***, p < 0.001; ****, p < 0.0001). The shift in axon sizes toward smaller to medium diameters in *oTbx1*^+/−^ compared to *oTbx1*^+/+^ mice resulted in a smaller average axon size for *oTbx1*^+/−^ mice (Mann-Whitney non-parametric tests, p = 7.48 × 10).**D**. Differences in the percentage of myelinated axons for *oTbx1*^+/−^ versus oTbx1+/+ mice across axon diameters (Kolmogorov-Smirnov, p = 3.59 × 10).

Densely packed axons were observed in the fimbria (Fig. 9A). To determine relative myelin thickness, we compared the ratio of the inner axon diameter to the outer fiber diameter (i.e., the g-ratio) (Fig. 9B). As was the case with constitutive *Tbx1* mice, the g-ratio plateaued at approximately 0.8, the ratio for optimal signal conductance in the brain (88). However, no differences were observed in g-ratios between o*Tbx1*^+/−^ mice and o*Tbx1*^+/+^ mice across the entire range of axon diameters.

We separately determined the number and proportion of axons of different diameters. The fimbria of o*Tbx1*^+/−^ mice contained more axons of ≥ 300 nm and < 800 nm diameters but fewer axons of > 1,200 nm and < 1,500 nm diameters compared to o*Tbx1*^+/+^ mice (Fig. 9C). Consequently, the curve for the relative proportions of axons (y-axis) versus axon diameter (x-axis) shifted to the left in o*Tbx1*^+/−^ mice, compared to o*Tbx1*^+/+^ mice (Fig. 9D). Consistent with g-ratios, myelin thickness was indistinguishable between o*Tbx1*^+/−^ mice and *oTbx1*^+/+^ mice across all axon diameters (**Figure S2**). Thus, conditional *Tbx1* heterozygosity initiated in oligodendrocyte precursor cells selectively shifted the relative proportion of small to medium myelinated axons in the fimbria but did not affect myelin thickness.

In summary, conditional *Tbx1* heterozygosity in the oligodendrocyte lineage shifted the proportion of myelinated axons to smaller sizes without affecting the thickness of myelin in the fimbria.

## Discussion

The objective of this study was to delineate the cellular origin(s) of the diverse behavioral and myelin phenotypes observed in constitutive *Tbx1* heterozygous mice(58–62). As some of these behavioral phenotypes are recapitulated by initiating *Tbx1* heterozygosity in neonatal progenitor cells (72) and neonatal neural progenitor cells in the subventricular zone are responsible for generating neurons and oligodendrocytes during the neonatal period (68, 69, 106), we reasoned that *Tbx1* deficiency in neonatal neural progenitor cells, neuronal lineage from neural progenitor cells, or oligodendrocyte lineage from oligodendrocyte precursor cells contributes to the observed myelin and associated behavioral phenotypes. This study aimed to investigate the impact of *Tbx1* heterozygosity in the oligodendrocyte lineage on the behavioral and ultrastructural phenotypes.

To achieve this objective, we crossed *Pdgfrα*Cre mice with congenic *Tbx1*^+/flox^ mice to generate *Pdgfrα*Cre;*Tbx1*^+/flox^ (o*Tbx1*^+/−)^ mice. We evaluated a range of social, cognitive, anxiety-related, and motor behaviors, along with the ultrastructural composition of myelin and axons in the fimbria. The o*Tbx1*^+/−^ mice selectively replicated the altered axon sizes in the fimbria observed in constitutive *Tbx1* heterozygous mice. In contrast, while o*Tbx1*^+/−^ exhibited improved spontaneous alternation following a 30-second delay at one month of age—indicative of enhanced working memory and cognitive flexibility–this was contrary to the impaired spontaneous alternation observed in constitutive *Tbx1* heterozygous mice. This observation supports that suggestion that the final phenotype arises from the cumulative effects of various contributory and opposing factors (54, 80).

Other phenotypes associated with constitutive *Tbx1* heterozygosity were absent in o*Tbx1*^+/−^ mice, including neonatal social communication, responses to social or non-social stimuli, and anxiety-like behaviors in an elevated plus maze and thigmotaxis. These findings indicate that *Tbx1* in the oligodendrocyte lineage does not significantly influence the phenotypes. Combined with the observation that *Tbx1* heterozygosity initiated in neonatal (P1–P5) neural progenitor cells at P1-P5, but not P21–P25, recapitulates the social and cognitive deficits observed in constitutive *Tbx1* heterozygous mice (72), the current negative data suggest that *Tbx1* in neonatal neural progenitor cells or their neuronal lineage may play a principal role in determining the final phenotype (72).

The fimbria of o*Tbx1*^+/−^ mice had more axons of ≥ 300 nm and < 800 nm diameter and fewer axons of ≥ 1,200 nm and < 1,500 nm diameter than o*Tbx1*^+/+^ mice. This was similar to the increase in axons of ≥ 200 nm and < 400 nm and the decrease in axons of ≥ 700 nm and < 1,700 nm axons in constitutive *Tbx1* heterozygous mice. The medium-size axons of constitutive *Tbx1* heterozygous mice have increased myelin thickness and lack large (≥ 1,700 nm) myelinated axons in the fimbria(62), but these characteristics were absent in o*Tbx1*^+/−^ mice. This selective change in the axon phenotype could result from widespread activities of the *Pdgfrα* promoter in many cell types and regions. Although *Pdgfrα*Cre induces recombination in oligodendrocyte precursor cells, it also triggers recombination in neurons, astrocytes, pericytes, ependyma, perivascular mesenchymal cells, and other cells(74–77, 97, 107, 108). *Tbx1* heterozygosity in neurons might underlie the shift in the axonal composition of neurons. The correlation between altered spontaneous alternation and the increased proportion of small-to-medium myelinated axons in the fimbria does not necessarily indicate causality by oligodendrocyte precursor cells. *Pdgfra*Cre-induced recombination could have affected phenotypes through other cell types and their progenies in other brain regions. In the mouse brain, *Tbx1* is not detectable in astrocytes or microglia; however, it is significantly present in endothelial cells and neural progenitor cells. Additionally, *Tbx1* can be detected in immature neurons as well as both excitatory and inhibitory neurons at various developmental stages, ranging from embryonic development to adulthood(70, 71, 109, 110). Further research is necessary to elucidate the effects of Tbx1 in different cell types on phenotypic outcomes.

Several interpretative limitations warrant attention. The general absence of phenotypes may be attributed to the high mortality rate of o*Tbx1*^+/−^ litters, potentially biasing our sample toward mice that were less affected by or recovered more quickly from the mutation’s effects. Among male pups in our breeder colony, only 31% and 24% survived to P7 and 2 months of age, respectively. However, two lines of evidence do not support this possibility. First, more mice were alive during the neonatal period than at 1 and 2 months of age, yet neonatal vocalizations on P8 and P12 were normal. Second, our nestinCreERT;*Tbx1*^+/flox^ litters also exhibited a high mortality rate but demonstrated more profound behavioral deficits (72).

We cannot exclude the possibility that the conditional *Tbx1* heterozygosity was incomplete. The *Pdgfrα* promoter induces nearly complete recombination in oligodendrocyte precursor cells, with labels persisting in their mature progeny oligodendrocytes (107, 111); however, this promoter may exhibit variability in some cells, leading to incomplete recombination (112). Furthermore, the lack of behavioral deficits may arise from compensatory processes in oligodendrocyte lineage cells; ablation of specific oligodendrocyte precursor cells did not result in gross behavioral abnormalities (66).

Large-scale human brain imaging studies show alterations in the white matter in CNV carriers(113, 114), as well as idiopathic cases of ASD and schizophrenia (115, 116). Moreover, carriers of hemizygous deletion of 22q11.2 have an altered white matter in the fornix/fimbria(117) and volume alterations of many brain regions(118–120). Together with our previous observations that *Tbx1* heterozygosity during embryonic neurogenesis (61) and in neonatal stem cells (72) likely contribute to volume alterations of specific brain regions and social and cognitive deficits (61), respectively, the present negative results delineate the extent to which *Tbx1* contributes to the behavioral and structural phenotypes of 22q11.2 hemizygosity through different cell types.

## Supplementary Material

1

This is a list of supplementary files associated with this preprint. Click to download.

- SupplementaryInformation.docx
- TableS3.pdf

## Data Availability

All data and materials are available upon request.

## List of abbreviations

CNVs: Copy number variations
ASD: Autism Spectrum disorder
scRNA-seq: single cell RNA-seq
Pdgfra: Platelet-derived growth factor receptor alpha
MAG: Myelin associated glycoprotein
MOG: Myelin oligodendrocyte glycoprotein
MBP: myelin basic protein
