# Supplementary material for: Tbx1 Heterozygosity in the Oligodendrocyte Lineage Shifts Myelinated Axon Composition in the Mouse Fimbria Without Behavioral Impairments
Source: Res Sq. 2026 Apr 19:rs.3.rs-9327970. Preprint. [Version 1] doi: 10.21203/rs.3.rs-9327970/v1 (PMC13105105; doi:10.21203/rs.3.rs-9327970/v1)
Supplement: 1 [file NIHPPRS9327970V1-supplement-1.pdf]

Supplementary Tables

Table S1

Primers used for genotyping.

Mouse line	Forward (5’ à 3’)	Reverse (5’ à 3’)	Purpose
Pdgfrα-Cre	olMR1084 (GCGGTCTGGCAGTAAAAACTATC)	olMR1085 (GTGAAACAGCATTGCTGTCACTT)	Generic cre - transgene
Pdgfrα-Cre	olMR7338 (CTAGGCCACAGAATTGAAAGATCT)	olMR7339 (GTAGGTGGAAATTCTAGCATCATCC)	Generic cre – internal positive control
ROSA-CAG-tdTomato	olMR9020 (AAGGGAGCTGCAGTGGAGTA)	olMR9021 (CCGAAAATCTGTGGGAAGTC)	tdTomato wild-type control
ROSA-CAG-tdTomato	olMR9103 (GGCATTAAAGCAGCGTATCC)	olMR9105 (CTGTTCCTGTACGGCATGG)	tdTomato mutant
Tbx1$^{+/flox}$	2g 1F (TCTTCTTGGGGCTGTAGACT)	Tbx1 1R (TGACTGTGCTGAAGTGCATC)	LoxP site
Tbx1$^{+/-}$	KO1F (TTGGTGACGATCATCTCGGT)	KO1R (ATGATCTCCGCCGTGTCTAG) Mut2R (AGGTCCCTCGAAGAGGTTCA)	Tbx1$^{+/+}$ Tbx1$^{+/-}$

Table S2

Primers for qRT-PCR

Gene	Assay ID
Tbx1	Mm00448949_m1
Cspg4	Mm00507257_m1
Mag	Mm00487538_m1
Mbp	Mm01266402_m1
Mog	Mm01279062_m1
Plp1	Mm01297210_m1
